# High Glucose-Mediated Oxidative Stress Impairs Cell Migration

**DOI:** 10.1371/journal.pone.0022865

**Published:** 2011-08-03

**Authors:** Marcelo L. Lamers, Maíra E. S. Almeida, Miguel Vicente-Manzanares, Alan F. Horwitz, Marinilce F. Santos

**Affiliations:** 1 Department of Morphological Sciences, Institute of Basic Health Science, Federal University of Rio Grande do Sul, Porto Alegre, Rio Grande do Sul, Brazil; 2 Department of Cell and Developmental Biology, Biomedical Sciences Institute, University of São Paulo, São Paulo, São Paulo, Brazil; 3 Department of Cell Biology, University of Virginia, Charlottesville, Virginia, United States of America; Roswell Park Cancer Institute, United States of America

## Abstract

Deficient wound healing in diabetic patients is very frequent, but the cellular and molecular causes are poorly defined. In this study, we evaluate the hypothesis that high glucose concentrations inhibit cell migration. Using CHO.K1 cells, NIH-3T3 fibroblasts, mouse embryonic fibroblasts and primary skin fibroblasts from control and diabetic rats cultured in 5 mM D-glucose (low glucose, LG), 25 mM D-glucose (high glucose, HG) or 25 mM L-glucose medium (osmotic control - OC), we analyzed the migration speed, protrusion stability, cell polarity, adhesion maturation and the activity of the small Rho GTPase Rac1. We also analyzed the effects of reactive oxygen species by incubating cells with the antioxidant N-Acetyl-Cysteine (NAC). We observed that HG conditions inhibited cell migration when compared to LG or OC. This inhibition resulted from impaired cell polarity, protrusion destabilization and inhibition of adhesion maturation. Conversely, Rac1 activity, which promotes protrusion and blocks adhesion maturation, was increased in HG conditions, thus providing a mechanistic basis for the HG phenotype. Most of the HG effects were partially or completely rescued by treatment with NAC. These findings demonstrate that HG impairs cell migration due to an increase in oxidative stress that causes polarity loss, deficient adhesion and protrusion. These alterations arise, in large part, from increased Rac1 activity and may contribute to the poor wound healing observed in diabetic patients.

## Introduction

Diabetes mellitus is a group of metabolic disorders that cause chronic hyperglycemia and is one of the most significant diseases in the developed world, affecting more than 170 million people. The tissue responses to diabetic conditions are varied; many are associated with oxidative stress in the cells [1]. The improper management of hyperglycemia leads to severe complications in diabetic patients: approximately 15% of patients display impaired wound healing, causing long-term complications such as limb amputation [2].

Skin wound repair involves a series of coordinated processes that include cell proliferation and migration, collagen deposition and remodeling, wound contraction, and angiogenesis. These processes involve different cell types, mostly fibroblasts/myofibroblasts, keratinocytes, and endothelial cells [3], [4]. While hyperglycemia has been linked to impaired wound healing, particularly altered angiogenesis and extracellular matrix remodeling [5], the nature of the linkage is unclear. Some studies have described alterations in cell migration associated with diabetic conditions. For example, Lerman et al. [6] showed that fibroblasts from diabetic mice migrate 75% less than those from normoglycemic mice and display a defective response to hypoxia, a condition commonly present in chronic wounds. A similar inhibition was recently observed in keratinocytes cultured in a high glucose environment [7], which suggests that high glucose plays a direct role on cell migration. However, none of these studies addressed the cellular mechanism by which this happens.

The migratory process is a cycle comprised of distinct, integrated steps that are regulated by the activation of signaling molecules. These steps are: polarization, in which the cell develops a clear front and rear; protrusion, which is driven by actin polymerization at the leading edge; the formation of substrate adhesions that serve to stabilize protrusions and generate the dynamic signaling, which converge on Rho GTPases. The cycle is completed with retraction at the cell rear and the release of adhesions [8]–[11]. The small Rho GTPases are central regulators that integrate and drive these processes; they act through several effector proteins that mediate migration. For example, Rac1 regulates the formation of the lamellipodium and adhesion dynamics, while RhoA is involved in the formation of actin bundles and adhesion maturation [10].

This study addresses the mechanism by which high glucose inhibits cell migration. We characterized the effect of an acute high glucose treatment on several migration-related parameters that define the steps of cell migration in different cultured cell types, including CHO.K1, NIH-3T3, mouse embryonic fibroblasts (MEFs). We have also used primary skin fibroblasts obtained from control and diabetic rats. We observed that high glucose increased reactive oxygen species (ROS) production, impaired cell polarization, decreased migration speed, protrusion persistence and stability, and adhesion maturation. These effects point to the Rho GTPases as mediators of these effects. In this regard, we observed a significant increase in the activation of the small GTPase Rac1, which is inhibited by antioxidants. Consistently, antioxidants reverted most of the migratory effects caused by high glucose. Together, our data indicates that hyperglycemia impairs cell migration through increased generation of ROS, which induces an abnormal activation of Rac1.

## Results

### High glucose decreases migration speed and directionality

**Figure 1:**
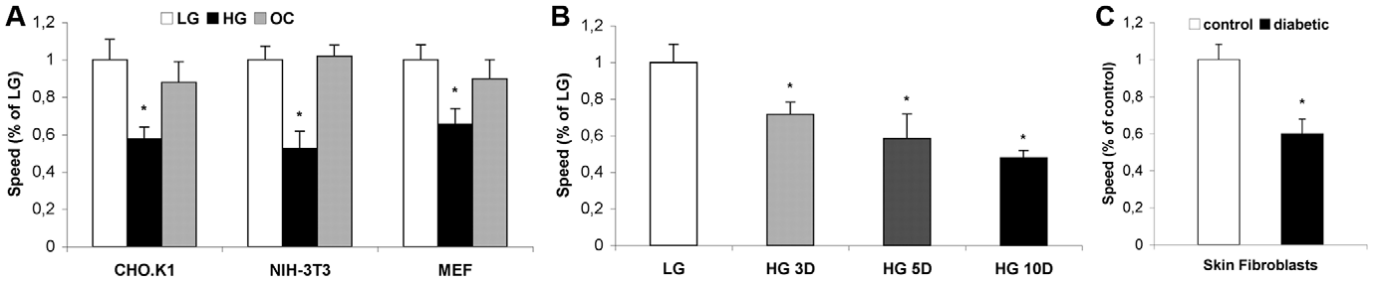
High glucose decreases migration speed in different cell types. A: Effects of 3 days treatment with low glucose (LG), high glucose (HG) or osmotic control (OC) medium on migration speed of CHO.K1, NIH-3T3 and MEF cells from at least 4 independent experiments. B: Effects of 3, 5 or 10 days with LG or HG medium on migration speed of MEF cells from at least 3 experiments. C: Migration speed of primary skin fibroblasts from control and diabetic rats. Results are shown as the % of the control (LG) ± SEM. (*) P≤0.01 according to Student's *t* test or One-way analysis of variance (ANOVA) followed by Tukey's post-test.

To determine the effects of high glucose treatment on cell migration, we treated CHO.K1, NIH-3T3 fibroblasts or mouse embryonic fibroblasts (MEF) with LG (low glucose), HG (high glucose) or OC (osmotic control) media for 3 days. We then plated the cells on 2 µg/ml fibronectin, which promotes cell migration [12], and imaged them using time-lapse microscopy. Acute treatment with HG decreased the migration speed by ∼40% compared to LG or OC cells (Figure 1A) (p<0.01, n = 50 cells). These effects are time-dependent; MEF migration was decreased by ∼60% after 10 days of treatment with HG medium compared to LG medium (Figure 1B, p<0.01, n = 20 cells). Similar results were obtained with primary fibroblasts from control and diabetic rats (Figure 1C, p<0.01, n = 20 cells). The decrease in migration speed caused by HG was also accompanied by a decrease in cell directionality, which likely contributes to the migration impairment (Figure S1). The fact that the OC group showed almost no effect on cell migration suggests that glucose internalization and metabolism are necessary for this effect on the migratory process.

### High glucose increases the number of cell protrusions but decreases their stability

**Figure 2:**
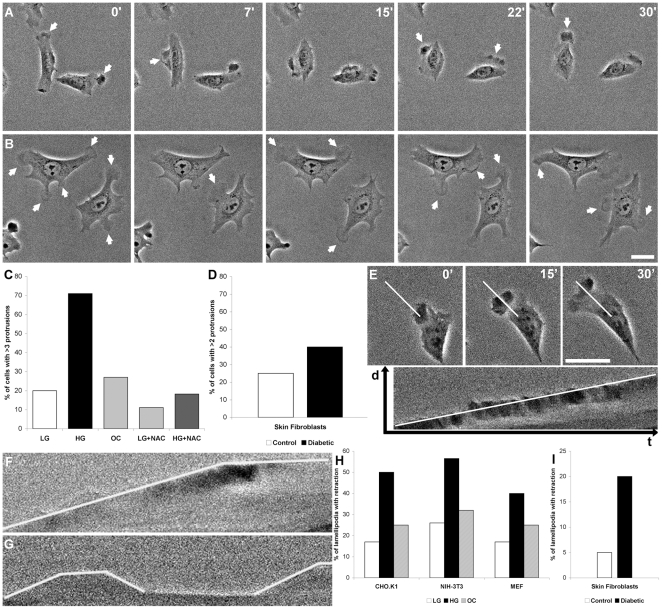
Cells cultured with high glucose show an increased number of short-lived protrusions. Photomicrographs show changes in CHO.K1 cell protrusions (arrows) within 7, 15, 22 and 30 minutes in cells cultured under control (A) and high glucose (B) conditions. C: Shows the percentage of CHO.K1 cells with more than 3 protrusions over a 30 minutes period in low glucose (LG), high glucose (HG) or osmotic control (OC) medium and in the presence or absence of the antioxidant N-acetylcysteine (NAC, 10 mM) for 1 hour. D: Shows the percentage of primary skin fibroblasts of control and diabetic rats with more than 2 protrusions over a 30 minutes period. E: Kymograph showing the extension of a protrusion over time. F: Kymographs of LG and (G) HG treated CHO.K1 cells, showing that protrusions under high glucose treatment are less stable and retract, followed by the formation of new protrusions at the same places. H: shows the percentage of lamellipodia that retract over a 30 min period in CHO.K1, NIH-3T3 and MEF cells and (I) shows the same for primary skin fibroblasts from control and diabetic rats. Bar: 10 µm.

To study the mechanism underlying the observed decrease in migration caused by HG conditions, we addressed the effect of high glucose on protrusion and adhesion. Using time-lapse imaging, we observed that HG medium induced a threefold increase in the number of CHO.K1 cells displaying more than three protrusions compared to the LG and the OC groups (Figure 2A, 2B and 2C, Movies S1 and S2). This behavior was also observed with the NIH-3T3 cells (100% increase, data not shown) and, to a lower extent, in primary rat fibroblasts (60% increase, Figure 2D).

A large fraction of the HG-induced protrusions appeared unstable, i.e. exhibited brief cycles of quick extension and retraction, and increased ruffling. To quantify this, we analyzed the protrusions by generating kymographs (Figure 2E) [13]. These revealed that most of the protrusions of the LG and OC-treated cells exhibited a homogenous behavior, defined by a period of linear and persistent progression followed by a period of quiescence (Figure 2F). Only a small fraction of these protrusions showed retraction (Figure 2H). Conversely, in the HG group, the protrusions also extended forward and became quiescent, but the quiescent periods were much shorter and in 50% of the protrusions, followed by intense retraction (Figure 2H). In many cases, the cycle repeated more than once in a particular region of the cell (Figure 2G). We observed a modest but significant increase in the protrusion rates of 3T3-NIH and MEF cells (data not shown). Skin fibroblasts from diabetic rats displayed a similar behavior, i.e., a 4-fold increase in retraction frequency when compared to control fibroblasts (Figure 2I). These data suggest that high glucose treatment leads to an increase in the number of non-productive protrusive events in the cell.

### High glucose decreases adhesion maturation

**Figure 3:**
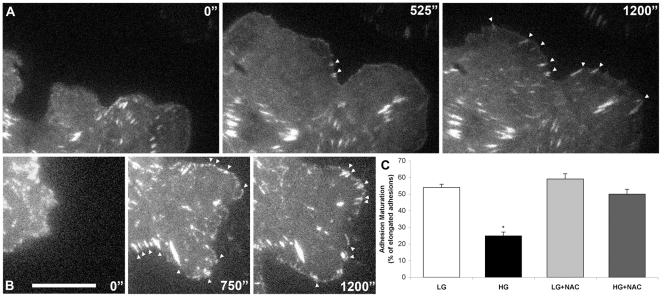
High glucose negatively affects adhesion maturation in CHO.K1 cells. After 2 days treatment, cells were transfected with GFP-paxillin and their adhesion dynamics analyzed 24 h later using TIRF microscopy. The increase in adhesion size and fluorescence intensity were considered signs of maturation (arrows). Time-lapse sequences show adhesions maturation of (A) high glucose (HG) and (B) Low glucose (LG) treated cells. C: The graph shows the probability of adhesion maturation of LG or HG treated cells, in the presence or absence of 10 mM N-acetylcysteine (NAC) for 1 hour, using at least 10 cells for each condition. Bar: 5 µm. (*) P≤0.01.

The relationship between protrusion stability and adhesion suggested that the observed alterations in protrusion might arise from defects in adhesion [9], [11]. To test this hypothesis, we expressed paxillin-GFP in cells in the LG and HG groups, and imaged their behavior when plated on fibronectin using total internal reflection microscopy (TIRF). LG medium promoted the formation of large, elongated adhesions, in approximately 55% of the adhesions present at the leading edge of CHO.K1 cells (Figure 3B and 3C, Movie S3). In contrast, only 25% of the adhesions in cells from the HG group underwent maturation (Figure 3A and 3C, Movie S4). These data suggest that decreased adhesion contributes to the observed protrusion instability in the HG group.

### High glucose activates the small GTPases Rac1 and RhoA

**Figure 4:**
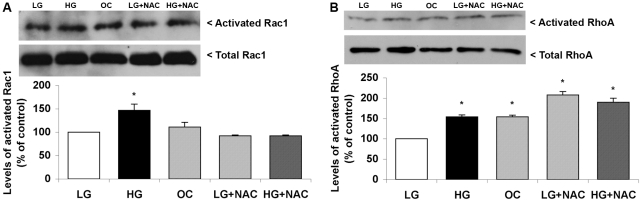
High glucose leads to Rac1 and RhoA activation in CHO.K1 cells. Representative western blotting showing the results of a pull-down assay for Rac1 activation and total Rac1 (A), as well as for RhoA activation and total RhoA (B) in control (LG), high glucose (HG) and osmotic control (OC), in the presence or absence of 10 mM N-acetylcysteine (NAC) for 1 hour. The ratio between activated and total proteins was measured by densitometry. Protein bands shown are representative of the experiments, and the results from 4 independent experiments were expressed as percentage of the control (LG) ± SEM. (*) P≤0.05 according to one-way analysis of variance (ANOVA) followed by Tukey's post-test.

Adhesion and protrusion are regulated by the Rho GTPases. Rac1 induces lamellipodial protrusion and adhesion formation, while RhoA mediates adhesion maturation and edge retraction [14], [15], [16]. To determine whether these GTPases underlie the observed effects of HG treatment in protrusion and adhesion, we determined the activation state of RhoA and Rac1 using an affinity pull-down system [17]. Both CHO.K1 and NIH-3T3 cells showed a moderate, but reproducible (40%), increase in GTP-bound (active) Rac1 compared to LG cells (Figure 4A, p<0.05, n = 5). HG also increased activated RhoA (Figure 4B, p<0.05, n = 3). Interestingly, increased osmolarity also increased RhoA, but not Rac1 (Figure 4B, p<0.05, n = 3). These results indicate that HG conditions promote activation of Rac1 due to a specific effect related to the glucose metabolism and not a mechanical effect caused by osmotic stress, which can, however, activate RhoA.

### High glucose alters cell polarity

**Figure 5:**
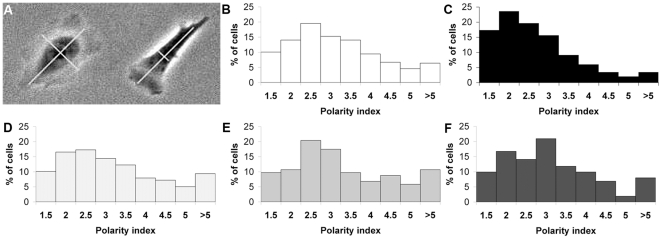
High glucose reduces morphological CHO.K1 cell polarity. A: The picture shows the two axes used for the polarity index calculation, with an example of a low polarized (left) and a highly polarized cell (right). The histograms show the distribution of cells cultivated with low glucose (B), high glucose (C), osmotic control (D), low glucose +10 mM N-acetyl-cysteine (NAC) for 1 h (E) and HG +10 mM NAC for 1 h (F), regarding polarity.

Altered GTPase activation often leads to aberrant cell morphologies. High Rac1 activation, in particular, can lead to large, round cells without a clear front and back [15]. To determine the effect of HG leads on cell polarity, we analyzed over >600 cells treated with each condition. We used a polarity index (PI), which is the length of a cell in the direction of migration (migratory axis) divided by its width orthogonal to the migration axis passing through the center of the nucleus (transverse axis) (Figure 5A). Most of the CHO.K1 cells of the LG and OC groups were elongated (Figures 5B and 5D, 2.5<PI<3.5; 49% (LG) and 44% (OC) of the cell population) or very elongated [index PI>3.5; 27% (LG) and 31% (OC)] with only a small percentage round [PI<2; 24% (LG) and 25% (OC)]. In contrast, the HG group (Figure 5C), showed a large decrease in the fraction of highly elongated cells and an increase in the fraction of round cells [41% of the cells displayed a round shape (PI<2), 44% an elongated shape (2.5<PI<3.5), and only 14% a very elongated shape (PI>3.5)]. This suggests that acute HG treatment induces loss of cell polarization, which is consistent with the increased activity of Rac1 and the projection of multiple lamellipodia observed in these conditions.

### Diabetes increases ROS generation

**Figure 6:**
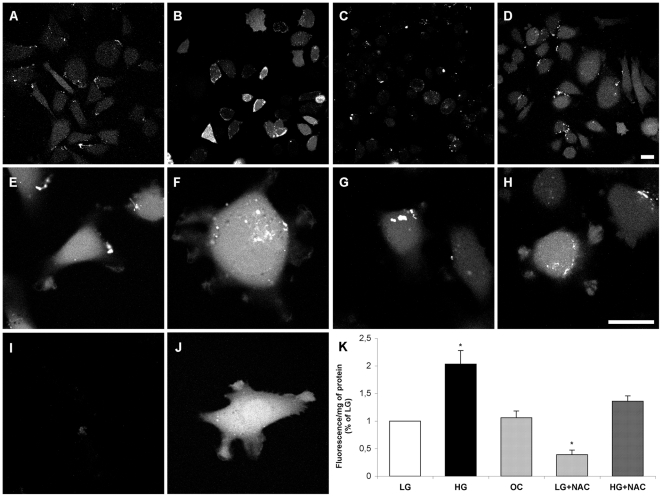
High glucose increases the generation of reactive oxygen species (ROS) in CHO.K1 cells. The probe 2′,7′-dichlorohydrofluorescein (DCFH_2-_DA) was employed in low glucose (LG) cells (A, E), high glucose (HG) cells (B, F), LG cells in the presence of 10 mM N-acetyl-cysteine (NAC) for 1 h (C, G), and HG cells in the presence of 10 mM NAC for 1 h (D, H). The negative control consisted of cells not exposed to the probe (I), while the positive control consisted of cells exposed to 10 µM H_2_O_2_ for 10 min (J). ROS were observed throughout the cell body and in cell protrusions, especially in high glucose-treated cells. K: Measurement of ROS using fluorimeter. OC = osmotic control. Results were expressed as percentage of the control (LG) ± SEM. (*) P≤0.01 using one-way analysis of variance (ANOVA) followed by Tukey's post-test. Bar: 10 µm.

Increased ROS generation is a hallmark of diabetes. To establish that high glucose increases ROS production in our system, we used an assay for ROS, using a fluorescence method, in cells treated for 3 days with LG or HG medium, both in the presence or absence of N-acetyl-cysteine (NAC, 10 mM, 1 h) [18], an antioxidant. Cells in HG medium exhibited a twofold increase in ROS compared to the LG and OC groups (Figure 6K); this increase was abolished by NAC treatment. Confocal microscopy confirmed the increase in ROS levels induced by HG treatment (Figure 6B and 6F); interestingly, most of the ROS localized to the cell body and protrusions, and both compartments exhibited elevated levels of ROS compared to the control groups (Figure 6A and 6E).

Glucose uptake and metabolism renders the cell more susceptible to increased ROS generation. Therefore, we assessed glucose metabolism in primary skin fibroblast cultures, and observed that cells from diabetic rats had a 40% increase in the expression of the glucose transporter GLUT1 (Figure S2A), which was also accompanied by a similar increase in glucose entrance into the glycolytic pathway (>40% increase on Hexokinase activity) and metabolism (35% increase on Pyruvate Kinase activity) (Figures S2B and S2C). Lactate dehydrogenase activity was not affected, suggesting that the increased glucose metabolism was mostly aerobic (Figure S2D).

### Decrease of ROS in HG cells improves migratory parameters

We then asked whether high glucose metabolism impairs cell migration through its effect on ROS generation. Treatment with NAC completely rescued the effect of HG medium on the number of protrusions per cell, reaching values similar to those obtained with LG or OC treatment (Figure 2C). Adhesion maturation in HG cells was also restored by NAC (Figure 3C). In addition, NAC also blocked the effect of high glucose on Rac1 activation (Figure 4A, p<0.05, n = 5). Interestingly, NAC did not inhibit the increase in RhoA activation; furthermore, it increased the degree of RhoA activation by 2-fold, even in LG cells (Figure 4B, p<0.01, n = 3). Finally, the depolarizing effect of HG treatment was also rescued by NAC (Figure 5F); importantly, NAC treatment had no effect on control cells (Figure 5E). This data indicates that ROS generation is involved in the inhibition of cell migration observed with HG treatment through a mechanism that involves the activation of Rac1 and its balance with the activation of RhoA.

## Discussion

Our data show that increased glucose uptake by fibroblasts inhibits cell migration through inadequate activation of the small GTPase Rac1, which depends on the oxidative state of the cell. Intracellular glucose is metabolized through a series of enzymatic reactions that are optimized by molecular oxygen and electron transport, which provides energy for ATP generation; ROS are byproducts of this process. There is strong evidence that excessive glucose increases ROS formation [1], [19], [20], which in turn overcomes the antioxidant capacity of the cell (oxidative stress). This has deleterious effects, including the non-specific oxidation of proteins and lipids, alterations in gene expression and perturbations of different signaling pathways [21], [22]. Our results indicate that ROS generation decreases cell migration by over-activation of the small Rho GTPase Rac1. ROS generation and Rac1 activation are therefore part of a positive feedback loop, as Rac1 increases ROS generation by activating the NADPH oxidase system [23]. Furthermore, activation of Rac1 by a specific guanine-nucleotide-exchange factor suffices to induce glucose uptake into skeletal-muscle cells [24], thereby contributing to its own glucose- and ROS-dependent activation.

In migrating cells, Rac1 is activated near the leading edge and is thought to drive protrusion [25], [26]. Therefore, the local activation of Rac1 near the leading edge may generate a local increase of ROS in this cellular region that oxidizes cysteine residues in different redox-sensitive targets [27], including signaling adaptors that can modulate the activation of small GTPases such as Rac1 or RhoA [28], [29]. On the other hand, sustained oxidative stress may affect these proteins differently, due to excessive oxidation [22]. Interestingly, increased Rac1 activity was also observed in cardiac fibroblasts from diabetic mice [30] and in endothelial cells [31]. Furthermore, inhibition of either Rac1 or NADPH oxidase activity protected from vascular injury in this system [31], [32]. Our data strongly suggest that most of the cellular effects of HG are due to a modest increase in Rac1 activation, which is in agreement with other studies that have shown that moderate Rac1 activation is sufficient to decrease migration speed and directionality, as well as to promote an increase in the number of protrusions per cell [33]. However, the increased Rac1 activity may lead to its mislocalization, which would amply the effect.

Our data also shows that HG increases RhoA activity. Rac1 and RhoA are reciprocally regulated by a negative feedback loop [17], [28], which may implicate NAPDH oxidase [21], [23]. However, the increase in RhoA activity is not specifically dependent on the glucose metabolism, since the osmotic control also displayed similar increase. Osmotic pressure applies mechanical stress to the cellular cortex, which may contribute to this activation similar to mechanical tension which, applied to the cell membrane, increases RhoA activation [34], bypassing the requirement of low Rac activity and thus breaking down the Rac-RhoA feedback loop. However, RhoA is also associated to the deleterious effects of diabetes, particularly in mesangial cells [35], [36], as well as in several organs that are targeted by diabetes [37]–[39].

The crosstalk between Rho GTPases and other pathways under HG conditions cannot be ruled out. For example, the inhibition of the nutrient-sensing mammalian target of rapamycin (mTOR) pathway inhibits RhoA and Rac1 activity, affecting cell motility [40]. This pathway is activated by glucose and, in several cell types (including fibroblasts), its activation is required for acquiring a senescent phenotype [41], [42]. One of the phenotypical alterations promoted by HG in fibroblasts was an increased cell size (Fig. 2), which is also observed in senescent cells, due to hypertrophy [43]. Interestingly, there is a positive feedback between the TOR pathway and ROS generation. ROS are generated in mitochondria in response to glucose, stimulating the mTOR pathway [44]. TOR signaling, on the other hand, may regulate mitochondrial proteome dynamics; it was shown that reduced TOR signaling increases mitochondrial oxygen consumption and decreases ROS generation [45]. Thus, the effect of NAC reversing the HG phenotype might be at least partially related to the inhibition of the mTOR pathway.

In summary, this study provides novel mechanistic insight into the effects of high glucose on cell migration, which is a likely contributor to the defects in wound healing often observed in diabetic patients. Our results provide a mechanistic framework, i.e. increased Rac1 activation, that explains previous observations.

## Materials and Methods

### Animals and primary cell culture preparation

All experiments were conducted in accordance to the NIH guidelines, and the protocols were approved by the Biomedical Sciences Institute/University of São Paulo Ethical Committee for Animal Research (2005, n109, fl10, lv2). Adult male Wistar rats were maintained in individual cages, with food and water available *ad libitum*. Six rats were used, with the same weight, equally divided in control and experimental groups. After a 12 h fasting, the experimental group was injected with streptozotocin (60 mg/kg body weight in 0.05 M citrate buffer, pH 4.5), while the control group received only the vehicle. Glycaemia was measured 24 h later and at the moment of the sacrifice, using Accu-Check® (Roche Diagnostics) and only animals with glycaemia higher than 300 mg/dl were considered diabetic. The body weight, food intake, blood glucose and urine volume were measured periodically. The glucose content in urine was also measured using Glucose HK Liquiform® (Labtest, Sete Lagoas, MG, Brazil). After 30 days, the animals were sacrificed with an overdose of ketamine and xylazine. All animals of the experimental group showed significant alterations in physiological parameters such as body weight, glycaemia and glycosury, confirming the diabetic state. When compared to the control group, diabetic animals did not gain weight and showed hyperphagia, high levels of blood glucose and glycosury (Table S1).

### Cell culture, conditions and transfection

CHO.K1 and NIH-3T3 cells were obtained from American Type Culture Collection (ATCC, Manassas, VA) while MEF were obtained at Gene Targeting and Transgenic Facility at University of Virginia (Charlottesville, VA). The cells were cultured under standard conditions, with DMEM low glucose medium (5 mM D-glucose) containing 10% fetal bovine serum (FBS), 100 U/ml penicillin and 1% non-essential amino acids (NEAA). For the primary skin fibroblast culture of control and diabetic animals, a 6 cm^2^ skin area was washed in PBS and the dermis was scraped and enzymatically digested with type I collagenase (0.1%) for 1 hour at 37°C under gentle agitation. After the addition of 5 ml FBS the cell suspension was centrifuged at 400 rpm for 10 min. The pellet was transferred to a Petri dish containing DMEM low glucose medium (for control fibroblasts) or DMEM high glucose medium (for diabetic fibroblasts), both containing 10% FBS, 100 U/ml penicillin, 1% NEAA and 2.5 µg/ml fungizone. The medium was changed thrice a week and cells were trypsinized at 80% confluence. Passages 5–8 were used for the cell migration experiments.

Cells were cultured for 3, 5, or 10 days in DMEM medium containing 5 mM D-glucose (low glucose - LG), 25 mM D-glucose (high glucose - HG), or 25 mM L-glucose (OC). When used, N-acetylcysteine at 10 mM was added for 1 h prior to the experiments. For GTPase activation assays, cells were cultured for 3 days in the designated serum-deprived (0.5% FBS) medium for 12 h and then stimulated with complete medium for 1 h before the experiments. The cells were transfected with the paxillin-GFP plasmid [12] using Lipofectamine (Invitrogen, Eugene, OR) following the manufacturer's instructions. The reagents for cell culture were purchased from Gibco (Invitrogen, Eugene, OR), while the chemicals were purchased from Sigma (St Louis, MO).

### Enzyme activity

Primary dermal fibroblasts were cultured in LG or HG medium, trypsinized, centrifuged and lysed with 100 µl of buffer containing Imidazol (50 mM, pH 7.2 at 4°C); the lysate was submitted to centrifugation and enzymatic activity was measured in the supernatant. For the Hexokinase (HK) activity, 20 µl of the supernatant were added to a buffer containing 50 mM Imidazol (pH 7.2), 5 mM MgCl_2_, 5 mM ATP, 1 mM glucose, 0.4 mM NADP and 0.3 U glucose-6-phosphate dehydrogenase; for the Pyruvate Kinase (PK) activity, 20 µl of the supernatant were added to a buffer containing 50 mM Imidazol (pH 7.2), 5 mmol/l MgCl_2_, 0.1 mol/l KCl, 1 mmol/l ADP, 0.15 mmol/l NADH, 2 mmol/l phosphoenolpyruvate and 0.9 U lactate dehydrogenase; for the Lactate Dehydrogenase (LDH) activity the supernatant was incubated with phosphate/pyruvate (50.0 mM) buffer for 5 min followed by the addition of 11.3 mM NADH. The reactions were monitored by changes in the absorption at 340 nm wavelength for 10 min at 30°C using a spectrophotometer (Beckman DU-68 Beckman, Fullerton, CA); HK and PK activities were analyzed due to the formation of NADPH and LDH activity by the rate of NADH oxidation. One unit (U) of enzyme activity corresponds to the amount of enzyme that converts 1 µmol of substrate per min. The results were normalized to the amount of protein presented in the supernatant and expressed as U/mg protein. All the chemicals were purchased from Sigma (St. Louis, MO, USA).

### Microscopy and image processing

Cells were plated on fibronectin–coated glass-bottomed dishes (2 µg/ml for CHO.K1 and 1 µg/ml for NIH-3T3, MEF and primary fibroblasts) in CCM1 medium for 1 h and maintained at 37°C at pH 7.4 (migration promoting conditions). For phase microscopy, time-lapse images were captured at 10 min intervals (0.25 NA CFI Achro DL10× Nikon objective) with a charge-coupled device camera (Orca II; Hamamatsu Photonics, Iwata-City, Japan) attached to an inverted microscope (TE-300; Nikon, Tokyo, Japan) using Metamorph software (Universal Imaging Corp., Downingtown, PA).

Confocal images were collected on an Olympus FluoView 300 system (1.45 NA oil PlanApo 60× TIRFM objective). GFP was excited using the 488 nm laser line of an Argon laser (Melles Griot, Albuquerque, NM). A Q500LP dichroic mirror (Chroma Technology Corp. Rockingham, VT) and a HQ525/50 emission filter was used for GFP labeled cells. Fluorescence images were acquired using FluoView software (Olympus, Tokyo, Japan).

TIRF images were acquired in an Olympus IX70, inverted microscope (1.45 NA oil Olympus PlanApo ×60 TIRFM objective) fitted with a Ludl modular automation controller (Ludl Eletronic Products, Howthorne, NY) and controlled by Metamorph (Molecular Devices). GFP was excited using the 488 nm laser line of an Ar ion laser (Melles Griot). Also, a dichroic mirror (HQ485/30) and a HQ525/50 emission filter were used. All Images were acquired with a charge-coupled device camera (Retiga Exi; Qimaging, Surrey, Canada) and analyzed using ImageJ software (http://rsbweb.nih.gov/ij).

### Kymography

For kymography, images were captured every 5 s for 30 min (0.65 NA CFI Achro DL 40× Nikon objective). A line (5 pixels-wide) was drawn along regions oriented in the protrusion direction and perpendicular to the lamellipodial edge. Protrusion parameters were quantified by kymography [13], using Image J software. The results were plotted in a graph where the Y axis is the distance reached by the lamellipodium along that line, and the X axis is time.

### Rho and Rac activation assay

Pull-down assays for activated Rac1 and RhoA were performed as described previously [46]. Briefly, after overnight serum starvation, the cells were stimulated with serum for 1 h, washed and lysed in CRIB buffer containing 1% NP-40, 50 mM Tris pH 7.4, 10% glycerol, 100 mM NaCl, 2 mM MgCl_2_, a protease inhibitor cocktail (Sigma P8340) and 20 µg of recombinant GST–PBD (Rac1) or GST-Rhotekin (RhoA). Cell lysates were then incubated with glutathione–agarose beads (Pharmacia, Stockholm, Sweden) for 30 min at 4°C, washed with lysis buffer and eluted with SDS sample buffer. Bound Rac1 and RhoA were analyzed by Western blotting. Whole-cell lysates were also analyzed for the presence of Rac1 and RhoA for normalization.

### Western blotting

Samples were submitted to SDS-PAGE with 15% gels and transferred to a PDVF membrane. Deffated milk or 5% BSA in PBS/0.5% Tween 20 were used for blocking during 45 min at room temperature. Incubation with the primary antibodies anti-Rac1 (1∶1000, BD Biosciences, Franklin Lakes, NJ), anti-RhoA (1∶500, Santa Cruz Biotech, Santa Cruz, CA) or anti-GLUT1 (1∶1000, Abcam, Cambridge, MA) was performed overnight at 4°C. After washing, membranes were incubated with peroxidase-conjugated secondary antibody (Amersham Pharmacia Biotech., Chalfont St. Gilles, United Kingdom), and the reaction was detected by chemioluminescence (Pierce, Thermo, Rockford, IL).

### Detection of Reactive Oxygen Species (ROS)

Cells were cultured for 3 days with the specified medium, trypsinized, washed and incubated with 10 µM 5–6-chloromethyl-2′,7′-dichlorodihydrofluorescein diacetate, acetyl ester (CM-H_2_DCFDA, Invitrogen, Eugene, OR) for 15 min. Cells were lysed, centrifuged (1 min, 13000 rpm at 4°C), the supernatant was analyzed by fluorimetry (excitation 492 nm and emission 525 nm, Fluorocount Packard, Perkin Elmer, Walthan, MA), and the protein concentration quantified. As positive control the cells were incubated with 100 µM H_2_O_2_, and the probe was omitted for the negative control. Results were calculated by mg/protein and expressed as % of control. For ROS visualization, cells were plated on 2 µg/ml fibronectin-coated glass-bottomed dishes, incubated using the same method described above, and then viewed on the confocal microscope.

### Assessment of cell polarity

For assessment of cell polarity, the polarity index was calculated as the length of the major migration axis (parallel to the direction of movement) divided by the length of the perpendicular axis that intersects the center of the cell nucleus.

### Statistical analysis

Student *t* test and one-way analysis of variance (ANOVA) followed by Tukey's post-test were employed, and differences were considered significant when p≤0.05.

## Supporting Information

Figure S1 Migration of CHO.K1 cells cultured under low glucose (LG), high glucose (HG), and in osmotic control (OC) medium. Due to the lower rate and directionality, the distances traveled by HG cells are shorter when compared to the controls. Individual cell trajectories starting at the same point are shown: the shorter distances (<30 radials) in red, intermediate distances (30–60 radials) in blue, and longer distances (>60 radials) in black. The lower graph (B) shows the distribution of cells in each category.
(TIF)

Figure S2 Diabetes increases glucose metabolism in dermal fibroblasts. Expression of the glucose transporter GLUT1 (A) and the activities of the glycolytic pathway enzymes Hexokinase (B), Pyruvate kinase (C) and Lactate Dehydrogenase (D) in primary skin fibroblasts of control and diabetic rats. Results were expressed as U/mg of protein and are shown as the % of the control ± SEM, n = 4 animals/group. The enzymatic activities were performed in triplicate. Proteins bands are representative of the experiment. (*) P≤0.01 according to Student's *t* test.
(TIF)

Table S1 Changes in physiological parameters after 30 days of induction of diabetes with streptozotocin.
(DOCX)

Movie S1 movie shows protrusions of CHO.K1 cells cultured with low glucose (control) during 3 days and plated under migration promotion conditions. This movie corresponds to Figure 3A. Total time = 30 minutes.
(MOV)

Movie S2 movie shows protrusions of CHO.K1 cells cultured with high glucose during 3 days and plated under migration promotion conditions. Cells produce several instable protrusions in different directions, when compared to control cells (Movie S1). This movie corresponds to Figure 3B. Total time = 30 minutes.
(MOV)

Movie S3 TIRF microscopy of dynamic adhesions of CHO.K1 cells transfected with GFP-paxillin, cultured under low glucose conditions (control). The increase in adhesion size and on fluorescence intensity were considered signs of maturation. This movie corresponds to Figure 4B. Total time = 20 minutes.
(MOV)

Movie S4 TIRF microscopy of dynamic adhesions of CHO.K1 cells transfected with GFP-paxillin, cultured under high glucose conditions. The increase in adhesion size and on fluorescence intensity were considered signs of maturation. Compared to control conditions (Movie S3), the number of mature adhesions in high glucose-treated cells is smaller. This movie corresponds to Figure 4A. Total time = 20 minutes.
(MOV)
