# Supplementary figures and images for: High Glucose-Mediated Oxidative Stress Impairs Cell Migration
Source: PLoS One. 2011 Aug 3;6(8):e22865. doi: 10.1371/journal.pone.0022865 (PMC3149607; doi:10.1371/journal.pone.0022865)

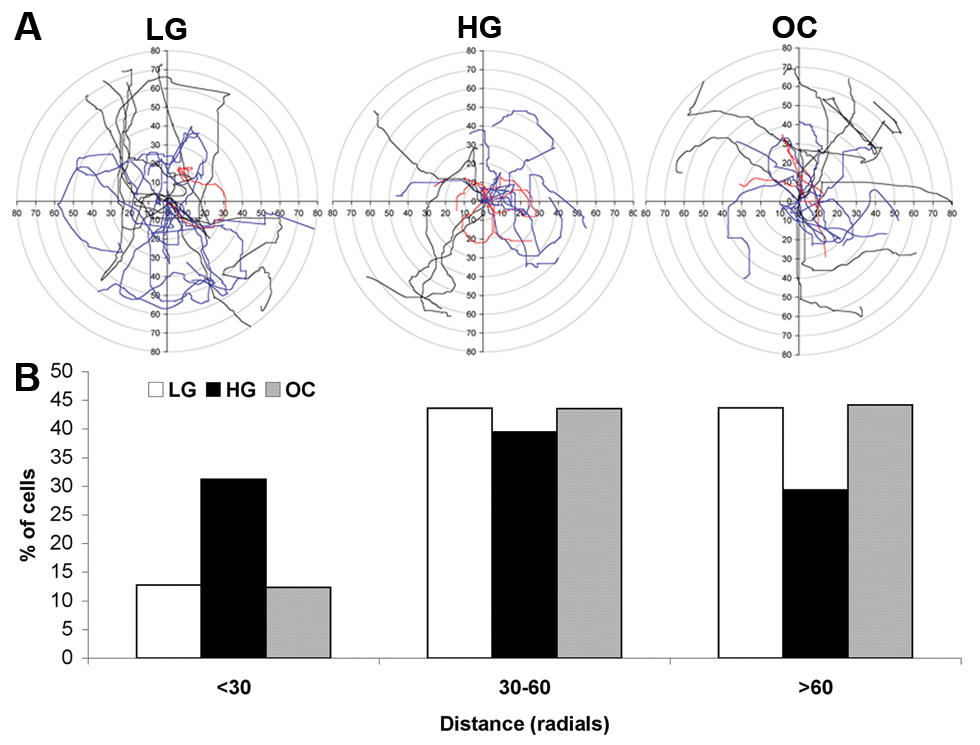

Supplement: Figure S1 — Migration of CHO.K1 cells cultured under low glucose (LG), high glucose (HG), and in osmotic control (OC) medium. Due to the lower rate and directionality, the distances traveled by HG cells are shorter when compared to the controls. Individual cell trajectories starting at the same point are shown: the shorter distances (<30 radials) in red, intermediate distances (30–60 radials) in blue, and longer distances (>60 radials) in black. The lower graph (B) shows the distribution of cells in each category. (TIF) [file pone.0022865.s001.tif]

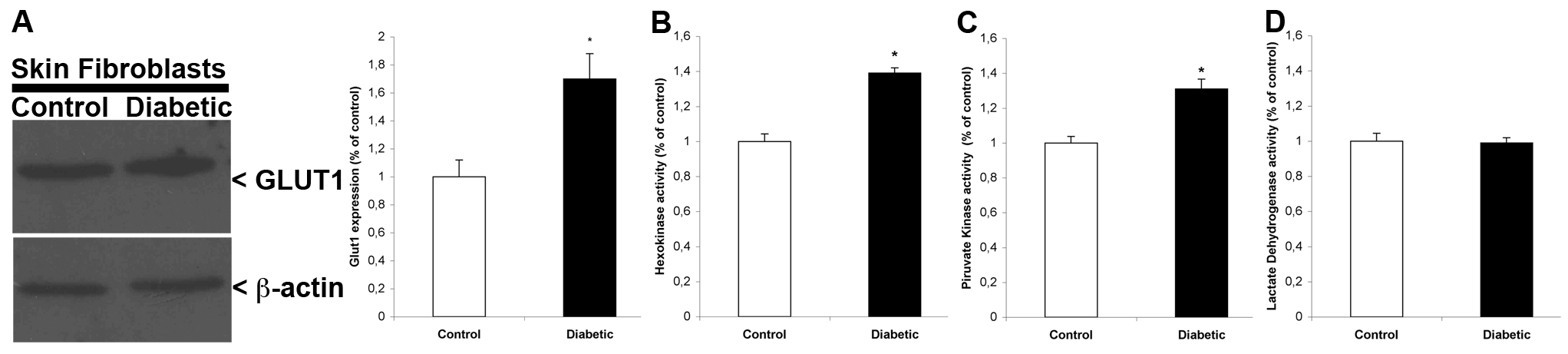

Supplement: Figure S2 — Diabetes increases glucose metabolism in dermal fibroblasts. Expression of the glucose transporter GLUT1 (A) and the activities of the glycolytic pathway enzymes Hexokinase (B), Pyruvate kinase (C) and Lactate Dehydrogenase (D) in primary skin fibroblasts of control and diabetic rats. Results were expressed as U/mg of protein and are shown as the % of the control ± SEM, n = 4 animals/group. The enzymatic activities were performed in triplicate. Proteins bands are representative of the experiment. (*) P≤0.01 according to Student's t test. (TIF) [file pone.0022865.s002.tif]
