# Supplementary material for: High Glucose-Mediated Oxidative Stress Impairs Cell Migration
Source: PLoS One. 2011 Aug 3;6(8):e22865. doi: 10.1371/journal.pone.0022865 (PMC3149607; doi:10.1371/journal.pone.0022865)
Supplement: Table S1 — Changes in physiological parameters after 30 days of induction of diabetes with streptozotocin. (DOCX) [file pone.0022865.s003.docx]

Supplemental Table 1: Changes in physiological parameters after 30 days of induction of diabetes with streptozotocin

|  | ∆ Body Weight (g) | Glycemia (mg/dl) | Glycosury (mg/day) |
| --- | --- | --- | --- |
| Control | 156±25,1 | 125±15,3 | 5±2 |
| Diabetic | 78,3±6,2* | 586,7±18,9** | 1445,9±12,4** |

Parameters of control and diabetic rats. Student’s T-test was used for statistical analysis. (*) = p<0.05 and (**) = p<0.001 when compared to control.
